# Long-Acting Antibiotics: New Opportunities Beyond Acute Bacterial Skin and Skin Structure Infections (ABSSSIs)!

**DOI:** 10.3390/antibiotics14020164

**Published:** 2025-02-07

**Authors:** Emanuele Pontali, Giammarco Baiardi, Filippo Del Puente, Francesca Mattioli

**Affiliations:** 1 Infectious Disease Unit, EO Ospedali Galliera, 16128 Genoa, Italy; filippo.del.puente@galliera.it; 2 Laboratory Medicine Unit, Department of Laboratory Diagnostics, IRCCS Ospedale Policlinico San Martino, 16132 Genoa, Italy; giammarco.baiardi@hsanmartino.it; 3 Pharmacology and Toxicology Unit, Department of Internal Medicine, University of Genoa, 16132 Genoa, Italy; francesca.mattioli@unige.it; 4 Clinical Pharmacology Unit, EO Ospedali Galliera, 16128 Genoa, Italy

**Keywords:** oritavancin, dalbavancin, lipoglycopeptides, endocarditis, prosthetic joint infection

Currently, two long-acting antibiotics are available: oritavancin (ORI) and dalbavancin (DBV). They are both lipoglycopeptides, derived from vancomycin and teicoplanin, respectively. They share several pharmacokinetic/pharmacodynamic (PK/PD) properties, such as a long elimination half-life; an antimicrobial spectrum restricted to Gram-positive bacteria only; acting by disrupting cell wall synthesis; a large distribution volume; optimal penetration in several tissues; intracellular penetration; slow, predominantly renal elimination; no clinically significant drug–drug interactions; and limited occurrence of adverse events [1,2].

They also share an approved label indication for the treatment of acute bacterial skin and skin structure infections (ABSSSIs) caused by Gram-positive organisms in adults and pediatric patients aged 3 months and older [1,2]. A single administration is considered to be active against pathogens for about two weeks [1,2]. Table 1 summarizes the key characteristics of the two drugs that justify their approved use and show the reasons for the growing interest in their use for other clinical indications [3,4,5,6,7,8,9,10,11].

Indeed, there is increasing experience in their use in different clinical settings where their peculiar PK/PD characteristics can be effectively exploited. As an example, when repeating DBV administration (1500 mg full dose) one week after the first dose, its activity is significantly prolonged by up to 6–8 weeks. When needed, further 1500 mg DBV doses can be administered at intervals between 3 and 6 weeks, thus providing a strategy for prolonged or suppressive treatments [12,13]. In contrast, less information is available for ORI [2]. Nevertheless, several reports exist on repeated weekly (or with longer intervals) administrations of various ORI doses for prolonged or suppressive treatments [2,14].

The clinical conditions which may require the repeated administration of the two drugs include but are not limited to osteomyelitis, prosthetic joint infections (PJIs), endocarditis (native or prosthetic valves), catheter-related infections, vascular infections, pediatric infections, and infections in diabetic patients [2,14,15,16,17,18,19,20,21,22,23]. Lipoglycopeptides’ bactericidal activity makes them even more interesting in the long-term treatment of such infections, especially when source-control surgery is not feasible or cannot be resolutive. Another intriguing property is their capacity to disrupt and penetrate biofilm. In fact, infections due to biofilm-producing bacteria (e.g., S. aureus, E. faecalis, etc.), particularly when they are involved in infections on prosthetic material, present significant challenges to obtaining a cure after antibiotic treatment. Thus, successful experiences support the use of lipoglycopeptides in this setting, i.e., when treating infections related to implanted materials, such as implantable cardioverter defibrillators (ICDs), short- or long-term vascular catheters, vascular prostheses, prosthetic valve infections (PVIs), PJIs, etc. [2,14,24,25,26,27].

**Table 1:** Clinical and pharmacological characteristics of long-acting antibiotics.

Characteristic		Oritavancin	Dalbavancin
Susceptible microorganisms		Gram+, including MRSA and VRE (including both Van A and B)	Gram+, including MRSA and VRE (Van B only)
Biofilm activity		Yes	Yes
Accumulation in macrophages		Yes	No
Pharmacokinetics	Adsorption	None by oral route, intravenous infusion	None by oral route, intravenous infusion
	Distribution	Exceeds total body water (>0.7 L/Kg); approximately 0.8 L/kg (V_d_), 85% protein-bound	Similar to the volume of extracellular fluid (0.2–0.7 L/Kg) (V_d_), 93% protein-bound
	Metabolism	No major metabolites; weak inhibitor or inducer of cytochrome P450 isoenzymes	Hydroxy–dalbavancin and mannosyle aglycone– dalbavancin (<25% of the administered dose)
	Elimination	Renal, slowly and unchanged (<1% fecal over 2 weeks after dosing) (393 h T_1/2_)	Renal (~10% as hydroxy–dalbavancin) and fecal (20% of administered dose) (372 h T_1/2_)
Pharmacodynamics		Inhibition of bacterial wall synthesis, permeabilization of bacterial membrane	Inhibition of bacterial wall synthesis, permeabilization of bacterial membrane
PK/PD parameter correlating with efficacy		In vitro *f*C_max_/MIC [7] In vivo *f*C_max_/MIC_,_ (*f*T > MIC comparable relationship) [8,9]	In vitro *f*T > 4 × MIC [3] In vivo *f*AUC/MIC not definitively identified *
Standard label dosing		1200 mg as a single intravenous infusion (lasting at least 3 h); new formulation with faster infusion becoming gradually available (single infusion in at least one hour)	1500 mg as a single intravenous infusion (lasting at least 30 min) or 1000 mg followed by 500 mg one week apart
Dose adjustments		No change with mild–moderate decrease in renal function or hepatic impairment	Required for severe renal impairment (creatinine clearance < 30 mL/min) Not required for renal replacement therapy
Potential for suppressive therapy		Yes	Yes
Multiple doses possible		Yes	Yes
Multidose off-label dosing in adults		1200 + 800 weekly or 1200 + 1200 weekly	3000 mg over 4 weeks with flexibility (preferred 1500 day 0 + 1500 mg day 7), then TDM guided with additional 1500 mg doses
Therapeutic drug monitoring		Not yet	Yes (not completely defined)
Good safety profile		Yes; caveat: alterations in coagulation tests	Yes
Infusion volume		1000 mL glucose 5% (250–500 mL glucose 5% or sodium chloride 0.9% for new formulation)	300–1500 mL glucose 5%

* Historically, the initial in vivo PK/PD index of *f*AUC/MIC derived for bacterial stasis, based on a neutropenic murine thigh model, has been substantially revised in the following years from the initial publication (*f*AUC/MIC > 265) [5] with a change of approximately 10-fold in a later publication (*f*AUC/MIC > 27.1) [6]. However, the earlier in vitro findings of dalbavancin time-dependent bactericidal activity at free drug concentrations equal to 4 × MIC values [3] were not taken into account when first testing dalbavancin PK/PD relationships in vivo [5].

In addition, DBV’s and ORI’s PK/PD properties make both viable alternatives, with limited outpatient administrations, to standard regimens of long-term treatment of Gram-positive bacterial infections consisting of antibiotics administered daily intravenously (IV) in both inpatient and outpatient settings. Patients’ quality of life would certainly improve with a reduction in hospital stay and/or subsequent accesses. Indeed, earlier discharge and shorter hospital stays are associated with improved quality of life, mobility, and the prevention of non-infectious and catheter-related patient complications [20]. Moreover, another specific area of use would be that of ‘difficult-to-treat’ patients, not due to disease, but due to socio-economic status (living far away from health centers, not having the economic support or the possibilities to travel to the health center daily, inability to access home care services, uninsured or partially insured patients, etc.), or difficulties in IV access for daily administration (e.g., previous or current IV drug use, previous chemotherapy, etc.) [27,28,29]. The reduced number of administrations would also prevent the positioning of major IV access points, such as central lines, peripherally inserted central catheters (PICCs), long-term central venous catheters (such as Porth-A-Cath); this would have an additional advantage, reducing the risk of healthcare-associated infections (HAIs) linked to catheter presence.

When using lipoglycopeptides repeatedly, a therapeutic drug monitoring (TDM)-guided approach could be a useful tool for optimizing the PK/PD relationship to avoid under- and overdosing which could lead to inefficacy and/or increased costs/toxicity [13,30,31]. This approach would be more impactful in special patient populations such as obese patients (unstudied so far) and vulnerable injecting drug users (IDUs), where opioid use, malnutrition, hypoproteinemia, cachexia, and altered tissue distribution may contribute to unpredictable blood levels and duration of efficacy [32]. Although information on how to determine DBV blood levels is currently widespread and many laboratories can achieve this [33,34,35,36], much less is known for ORI. For this latter drug, no validated quantitation method for the implementation of a TDM approach has been developed so far [2,19,27,28,30,31,37,38,39,40,41,42,43,44].

Furthermore, an additional advantage of these two drugs is their safety profile. Their excellent safety profile has also been confirmed in real-world studies evaluating the use of DBV in off-label settings (e.g., endocarditis, osteomyelitis, PJIs) or in systematic reviews for ORI [2,14,45].

Nevertheless, there are knowledge gaps that need to be addressed, such as the number of administrations needed in longer-term treatments, their timing and dosages to be given at each administration (for ORI: 800 or 1200 mg), the need for the association of other drugs in suppressive regimens, concentration ranges for TDM to decide on re-dosing (little is known for DBV [13,46,47], while nothing is known for ORI [2]), and the need to better define the PK/PD indexes for multidose regimens of both drugs.

In conclusion, long-acting antibiotics are increasingly being used with success beyond their original approved label indications [1,2,48]. Multidisciplinary research is necessary in order to address the unresolved issues and to provide support for the ‘off-label’ use of these two drugs.
